# Ochratoxin A induces immunotoxicity by targeting Annexin A1 mediated neutrophil apoptosis in zebrafish

**DOI:** 10.3389/fimmu.2025.1542964

**Published:** 2025-01-24

**Authors:** Yihong Zheng, Yinuo Liu, Jin Tian, Shuhong Liu, Gaowei Ma, Yupeng Xie, Chenhua Zheng, Zekai Wu

**Affiliations:** ^1^ Key Laboratory of Gastrointestinal Cancer (Fujian Medical University), Ministry of Education, School of Basic Medical Sciences, Fujian Medical University, Fuzhou, China; ^2^ Experiment Teaching Center of Basic Medical Sciences, School of Basic Medical Sciences, Fujian Medical University, Fuzhou, China

**Keywords:** ochratoxin A (OTA), immunotoxicity, apoptosis, Annexin A1, aesculetin

## Abstract

**Introduction:**

Ochratoxin A (OTA) is a toxic secondary metabolite produced by Aspergillus and Penicillium species, posing a significant threat to global food safety. Previous studies have demonstrated the diverse toxic effects of OTA, including hepatotoxicity, nephrotoxicity, and carcinogenicity. However, limited understanding exists regarding its immunotoxicity and the underlying mechanisms, particularly in relation to innate immunity.

**Methods:**

Zebrafish embryos were exposed to varying concentrations of OTA to assess its impact on embryonic development, innate immune cell formation, and immune response. Transcriptome sequencing analysis was performed to identify changes in gene expression. Additionally, the potential therapeutic effect of aesculetin was evaluated.

**Results:**

Our results demonstrated that exposure to OTA inhibited embryonic development and induced malformations in a concentration-dependent manner. Additionally, OTA exposure led to a significant reduction in the number of neutrophils and macrophages, indicating compromised formation of innate immune cells. Furthermore, OTA exposure hampered the immune response during zebrafish fin regeneration, as evidenced by the diminished migration of neutrophils and macrophages to the wound area. Transcriptome sequencing analysis identified significant up-regulation of the anxa1a and anxa1d-mediated apoptosis signaling pathway in neutrophils following OTA treatment. Notably, administration of aesculetin, known for its anti-apoptosis activity, effectively attenuated the immunotoxic effects induced by OTA.

**Discussion:**

These findings provide valuable insights into the immunotoxicity of OTA while highlight the potential therapeutic strategy using aesculetin for mitigating immune dysfunction caused by OTA.

## Introduction

Ochratoxin, a toxic secondary metabolite produced by *Aspergillus* and *Penicillium* species, comprises ochratoxin A (OTA), ochratoxin B (OTB) and ochratoxin C (OTC). Among these, OTA exhibits the highest toxicity. It is widely distributed across various sources, including cereals and cereal products (1). Additionally, it has been detected at varying levels in fruits, vegetables, meat products, dairy products, and animal feed (2). The contamination of food and medicinal materials with OTA can lead to acute or chronic toxicity upon ingestion into the human body (3). Consequently, the contamination of OTA has emerged as a significant threat to global food safety (4).

The metabolism of OTA primarily occurs in the liver through enzymatic processes, and its derivatives have been widely detected in plasma, milk, and urine samples of individuals who have consumed contaminated food (5). Animal studies have demonstrated that OTA exhibits a diverse range of toxic effects encompassing hepatorenal toxicity, reproductive and developmental toxicity, immunotoxicity, as well as carcinogenicity (6). The kidney and liver are predominantly affected, while immune cells in the thymus, spleen, and lymph nodes undergo structural and functional alterations, thereby influencing the function of immune organs (7). However, understanding of the mechanisms underlying OTA-induced immunotoxicity remains limited, particularly within the context of innate immunity.

Zebrafish is widely recognized as an excellent model organism for biotoxicology research (8). In comparison to other animal models, zebrafish exhibit rapid growth and development cycles, require smaller drug dosages, and are transparent during early development stages (9). Their morphology and internal structure can be directly observed under a microscope, significantly reducing the time required for animal experiments while also minimizing costs and experimental complexities. Currently, the complete genome of the zebrafish has been sequenced, with an 87% homology to human genome. Moreover, most of its gene sequences and immune system closely resemble those of humans (10). During the first three weeks after fertilization, larval zebrafish exclusively rely on their innate immune responses for survival, thereby providing a unique opportunity to investigate vertebrate innate immunity, excluding the potential influence of adaptive immune responses (11).

The immune system serves as the primary defense mechanism against pathogen invasion (12). It plays a crucial role in mediating responses to both biotic and abiotic stress signals (13). Immune activity is essential for maintaining internal homeostasis, including interactions with relevant microbiota (14). Immunity can be categorized into innate immunity and adaptive immunity based on the speed and specificity of response (15). The innate immune system primarily comprises neutrophils, macrophages, complement factors, cytokines, and acute-phase proteins that collectively provide timely host defense mechanisms (16). Neutrophils serve as the initial responders to infection by swiftly migrating to the pathogen invasion sites, therefore playing a pivotal role in effectively countering infections (17). The presence of chemokines induces the recruitment of macrophages to infection sites, where they engage in phagocytosis to engulf and subsequently eliminate or degrade foreign particles (18). Collectively, both neutrophils and macrophages play indispensable roles in innate immune responses.

In this study, utilizing zebrafish as a model organism, we found that OTA exerted suppressive effects on innate immunity. Exposure to varying concentrations of OTA inhibited the zebrafish embryonic development. Furthermore, the number of neutrophils and macrophages in caudal hematopoietic tissue (CHT) was decreased, and the migration of these immune cells to the wound area was impaired after caudal fin amputation. To elucidate the underlying mechanism of OTA immunotoxicity, we performed transcriptome sequencing on control and OTA-treated zebrafish embryos. By constructing a comprehensive molecular profile, we found a significant up-regulation of the *anxa1a* and *anxa1d*-mediated apoptosis signaling pathway in neutrophils following OTA treatment. Additionally, aesculetin treatment effectively restored the immunotoxic effects induced by OTA in zebrafish. In conclusion, this study uncovers a novel mechanism underlying OTA-induced immunotoxicity, and also proposes aesculetin as a potential therapeutic agent for mitigating OTA toxicity, thereby providing new insights into the diagnosis and treatment strategies for immune dysfunction related to OTA exposure.

## Methods

### Zebrafish husbandry

The wild type zebrafish (AB line) and transgenic lines *Tg(lyz:dsRed)* and *Tg(mpx:EGFP)* were obtained from the China Zebrafish Resource Center (CZRC, Wuhan, China). The fish were maintained in a circulating system at a temperature of 28 ± 0.5°C under a light/dark cycle of 14 hours light to 10 hours dark, and fed twice daily with freshly hatched brine shrimp, following the guidelines set by the Institutional Animal Care and Use Committee of Fujian Medical University.

### Chemical exposure

Healthy embryos at one day post-fertilization (1 dpf) were transferred into six-well plates and treated with OTA (TMRM, Changzhou, China) of 0 ng/mL, 10 ng/mL, 25 ng/mL, and 50 ng/mL. The mortality rates and malformation rates were recorded at 2 dpf and 3 dpf. Bright-field images were captured at 3 dpf using a stereomicroscopy (Nikon, Japan) after anesthetizing with 0.02% tricaine solution (Sigma-Aldrich, Germany). Body length and yolk sac area were measured with ImageJ software. The rescue group was treated with 20 μg/mL aesculetin (Must Bio-Technology, Chengdu, China) after treatment with 25 ng/mL OTA.

### Innate immunocyte analysis

The transgenic embryos of *Tg(lyz:dsRed)* and *Tg(mpx:EGFP)* lines at 3 dpf were used for neutrophil cell quantification. After anesthesia with 0.02% tricaine solution, fluorescence images were then captured using a stereo fluorescence microscope (Nikon, Japan) for quantification and analysis of neutrophil cells expressing dsRed or EGFP in the CHT of zebrafish embryos.

For macrophage analysis, a neutral red staining solution (final concentration of 2.5 mg/mL) (Yuanye Bio-Technology, Shanghai, China) was applied to zebrafish embryos at 3 dpf. The embryos were incubated in darkness at 28.5°C for three hours before being washed with phosphate-buffered saline (PBS) for thirty minutes. Images were captured using stereomicroscopy to quantify the number of positively stained cells in dorsal head.

### Caudal fin amputation and immunocyte migration assessment

Zebrafish embryos at 3 dpf were anesthetized and approximately 80% of the caudal fin was amputated using a surgical blade. Quantification of the neutrophils and macrophages at the wound area was performed at 4 hours post amputation, as described above.

### Oxidative stress analysis

Zebrafish embryos were stained with the DCFH-DA fluorescent probe (Beyotime, Shanghai, China) in darkness at a temperature of 28.5°C for thirty minutes. After rinsing with PBS, images were captured using a fluorescence stereomicroscope while maintaining consistent exposure settings throughout image acquisition across all groups. Subsequently, ImageJ software was utilized to quantify green fluorescence intensity for each group and calculate average fluorescence intensities.

### Quantitative real-time PCR

Total RNA of zebrafish embryos was extracted using the TRIzol reagent (Invitrogen, CA, USA) as previously reported (19). The RNA was reverse transcribed into cDNA using the Evo M-MLV reverse transcription kit (Accurate Biology, Changsha, China) according to the manufacturer’s instructions, and qRT-PCR was performed using the SYBR Green Pro Taq HS premixed qPCR kit (Accurate Biology, Changsha, China). The primer sequences used in this study are listed in **Table 1**. qRT-PCR was conducted on an Agilent AriaMX Real-Time PCR System (Santa Clara, CA, USA). Relative quantification of target gene expression levels was determined using the 2^-ΔΔCT^ method with zebrafish *actb2* gene serving as an internal reference.

**Table 1:** Primer sequences of qRT-PCR in zebrafish.

	Gene	Designed qRT-PCR primer sequences (5’ to 3’)	
		Forward	Reverse
Reference gene	*actb2*	CCCAAACCCAAGTTCAGCCA	ACCCACGATGGATGGGAAGA
Apoptosis-related genes	*anxa1a*	GACTTCAGGAATGCCCTGCT	TGGCGACGTCCACTTTACTG
	*anxa1d*	TCCTGAACGAGCAGCTTATCC	GCTCCTCTTTGCTAGCACCT
	*baxa*	TGGCAAGTTCAACTGGGGAA	ATAACTGCGGATTCCGTCCC
	*bcl2a*	AGATGGCGTCCCAGGTAGAT	AGAGTCTCTCTGCTGACCGT
	*casp3b*	ACAACACCAGAAGCAGGACTT	TTTGCATCGCTTTGTCTGGC

### Transcriptomic analysis

The total RNA extracted from control and 25 ng/mL OTA-treated zebrafish embryos was subjected to sequencing by Seqhealth Technology Co., Ltd. (Wuhan, China) using Novaseq 6000 (Illumina, CA, USA) with a read length of 150 base pairs. Differential expressed genes (DEGs) were analyzed using the DESeq2 package. Gene ontology (GO) and Kyoto Encyclopedia of Genes and Genomes (KEGG) analyses were performed utilizing the DAVID database. The mRNA expression patterns of *anxa1a* and *anxa1d* were visualized using Integrative Genomic Viewer (IGV) software. Sankey diagrams were generated via https://www.bioinformatics.com.cn. Gene set enrichment analysis (GSEA) was generated using https://www.gsea-msigdb.org/gsea/index.jsp.

### Molecular docking

The OTA structure was retrieved from the PubChem database (https://pubchem.ncbi.nlm.nih.gov). The crystal structures of ANXA1 were sourced from the RCSB Protein Data Bank (http://www.pdb.org). Docking simulations were conducted using AutoDock 4.2 software (20). During the docking process, water molecules and salts were removed, and polar hydrogen atoms and Gasteiger charges were added to the protein crystal structures through automated docking procedures. The Lamarckian genetic algorithm (LGA) was utilized for the docking procedures. The binding energy of the docked complexes was calculated using the Auto tool to assess the binding affinity between ANXA1 protein and OTA. The resulting complex was visualized using Pymol 2.5 software.

### Statistical analysis

The statistical analysis was conducted using GraphPad Prism version 9.0 software, and differences among groups were evaluated by one-way ANOVA for comparisons among the different groups. All results are presented as mean ± standard deviation (SD). Significance levels for differences across various treatments compared to the control group are indicated as p < 0.05 (*), p < 0.01 (**) or p < 0.001 (***).

## Results

### Developmental toxicity of OTA on embryonic zebrafish

**Figure 1:**
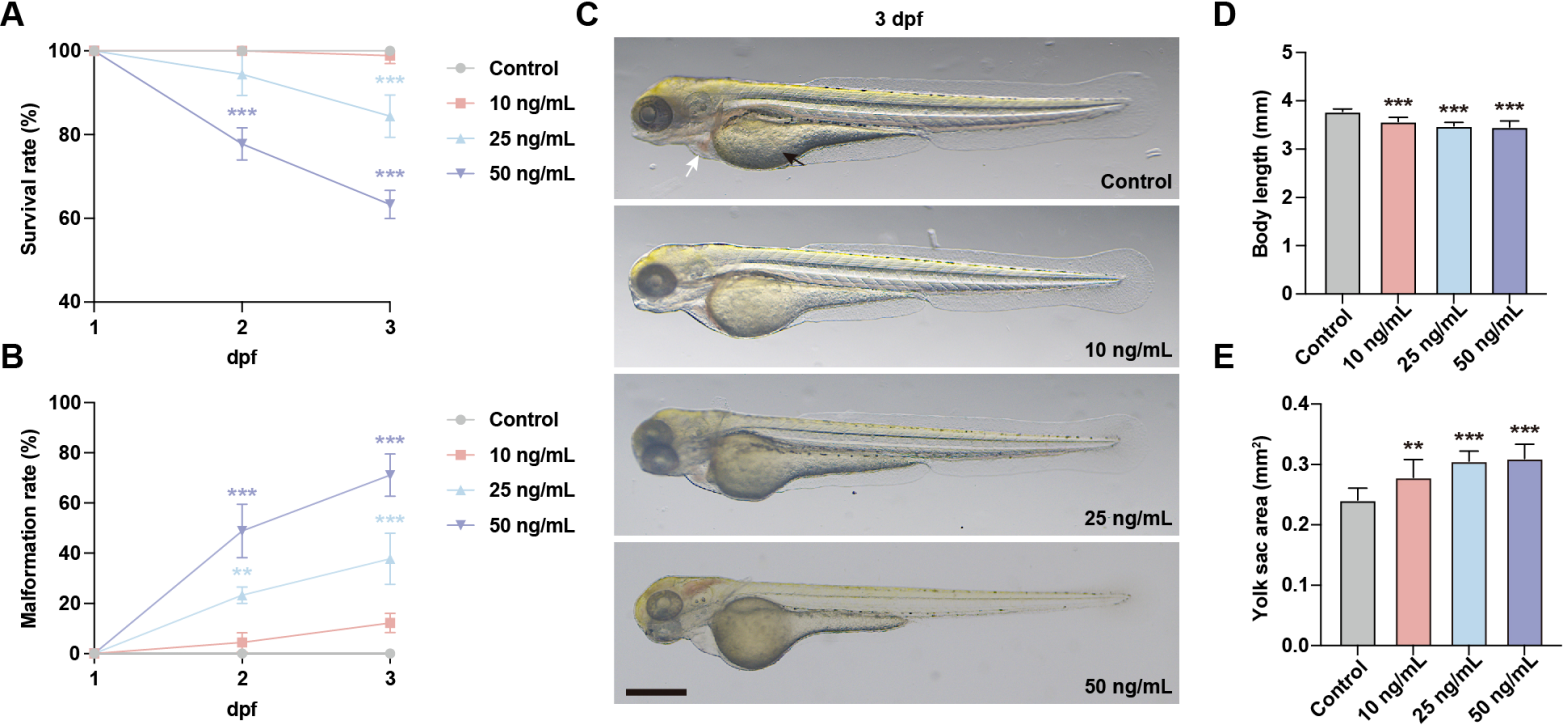
OTA induced developmental toxicity to embryonic zebrafish. **(A, B)** Survival rate **(A)** and malformation rate **(B)** of zebrafish larvae exposed to 0, 10 ng/mL, 25 ng/mL and 50 ng/mL OTA at 2 dpf and 3 dpf. One-way ANOVA-Dunnett test, **p < 0.01, ***p < 0.001. Error bar represents standard deviation. **(C)** Representative images of control and OTA-treated zebrafish larvae at 3 dpf. The black arrow represents yolk sac and the white arrow represents pericardial cavity. Scale bar, 200 μm. **(D, E)** Statistical analysis of the body length **(D)** and yolk sac area **(E)** of control and OTA-treated zebrafish larvae at 3 dpf. One-way ANOVA-Dunnett test, **p < 0.01, ***p < 0.001. Error bar represents standard deviation.

To investigate the general developmental toxicity of OTA exposure, zebrafish embryos at 1 day post fertilization (dpf) were subjected to OTA concentrations of 10 ng/mL, 25 ng/mL, and 50 ng/mL for 2 days. The results showed that the survival rate in embryos exposed to OTA at concentrations of 25 ng/mL and 50 ng/mL decreased in a dose-dependent manner. Specifically, after a period of 2 days’ exposure, the survival rates were inhibited by approximately 15.6% and 36.7%, respectively (**Figure 1A**). Furthermore, OTA-exposed embryos exhibited varying degrees of malformation, characterized by pericardial edema, reduced body length, and enlarged yolk sac area (**Figures 1B–E**). These findings indicated that OTA exerts developmental toxicity on zebrafish embryos in a concentration-dependent manner.

### OTA exposure impaired the development of innate immune cells

**Figure 2:**
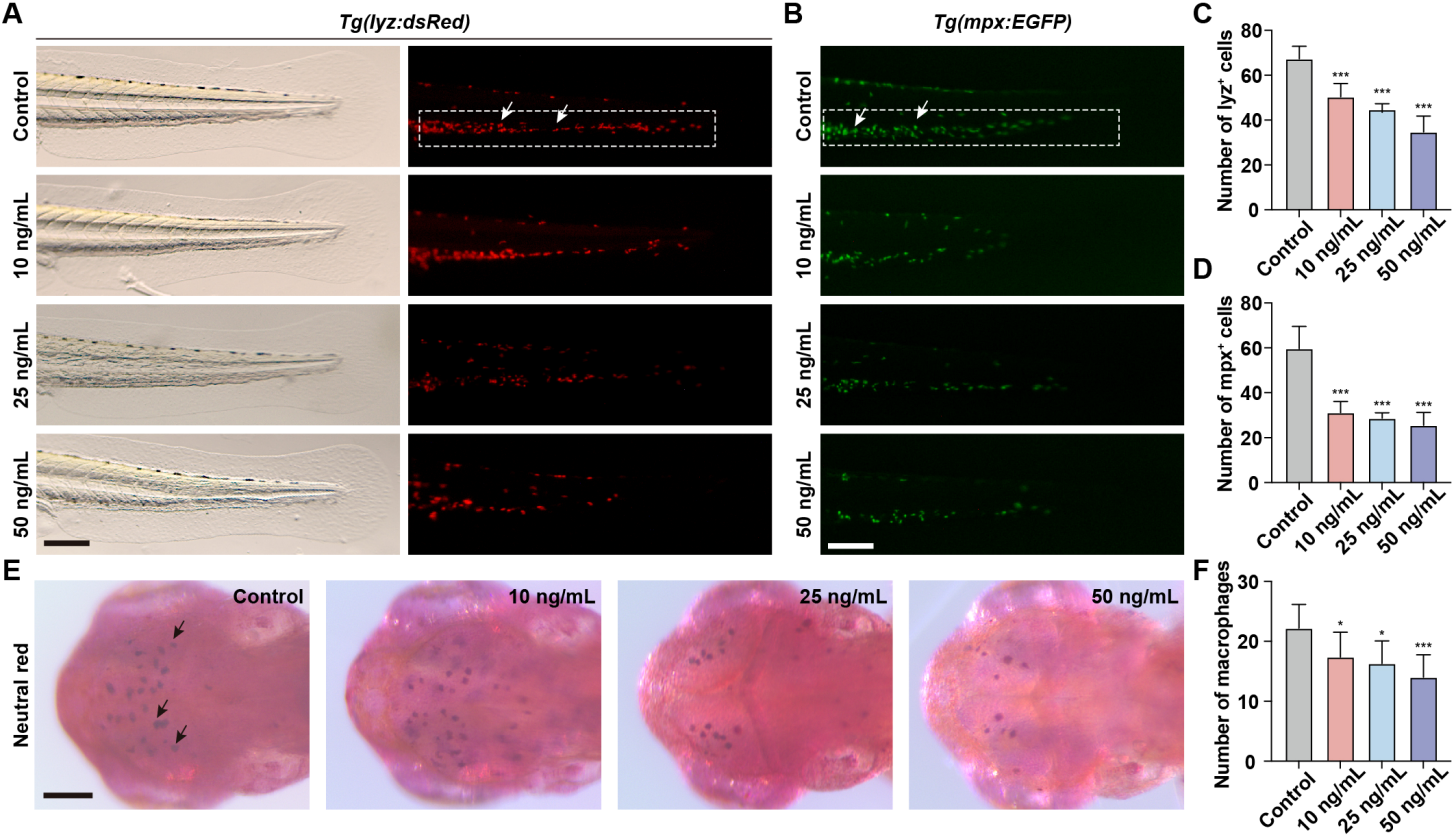
OTA impaired the formation of innate immune cells in zebrafish larvae. **(A, B)** Representative images of control and OTA-treated *Tg(lyz:dsRed)* **(A)** and *Tg(mpx:EGFP)* **(B)** transgenic zebrafish larvae at 3 dpf. The white dashed rectangle represents the CHT region and the white arrows represent the neutrophils. Scale bar, 100 μm. **(C)** Representative neutral red staining results of control and OTA-treated zebrafish larvae at 3 dpf. The black arrows represent neutral red labeled macrophages. Scale bar, 100 μm. **(D–F)** Statistical analysis of the number of neutrophils **(A, B)** and macrophages **(C)**. One-way ANOVA-Dunnett test, *p < 0.05, ***p < 0.001. Error bar represents standard deviation.

Neutrophils and macrophages, as integral components of the innate immune system, play a pivotal role in the immune response of zebrafish embryos. To assess the immunotoxicity associated with OTA exposure, we took advantage of *Tg(lyz:dsRed)* and *Tg(mpx:EGFP)* transgenic zebrafish lines to visualize neutrophils within zebrafish larvae. During the embryonic stage of zebrafish development, neutrophils are primarily located in the CHT. Therefore, quantifying the number of neutrophils in CHT serves as an indicator for neutrophil development. Following OTA treatment, there was a significant reduction in the number of neutrophils observed in the caudal region with increasing concentrations of OTA (**Figures 2A–D**). Macrophages within zebrafish larvae can effectively capture neutral red dye, resulting in their visualization as dark red cells. Consequently, by assessing changes in neutral red-stained macrophages within the head region, we also found that OTA exposure led to a dose-dependent decrease in macrophage numbers (**Figures 2E, F**). These findings collectively demonstrate that OTA impedes proper formation of innate immune cells and suggests compromised immune function within affected larvae.

### OTA exposure impaired the immune response during zebrafish fin regeneration

**Figure 3:**
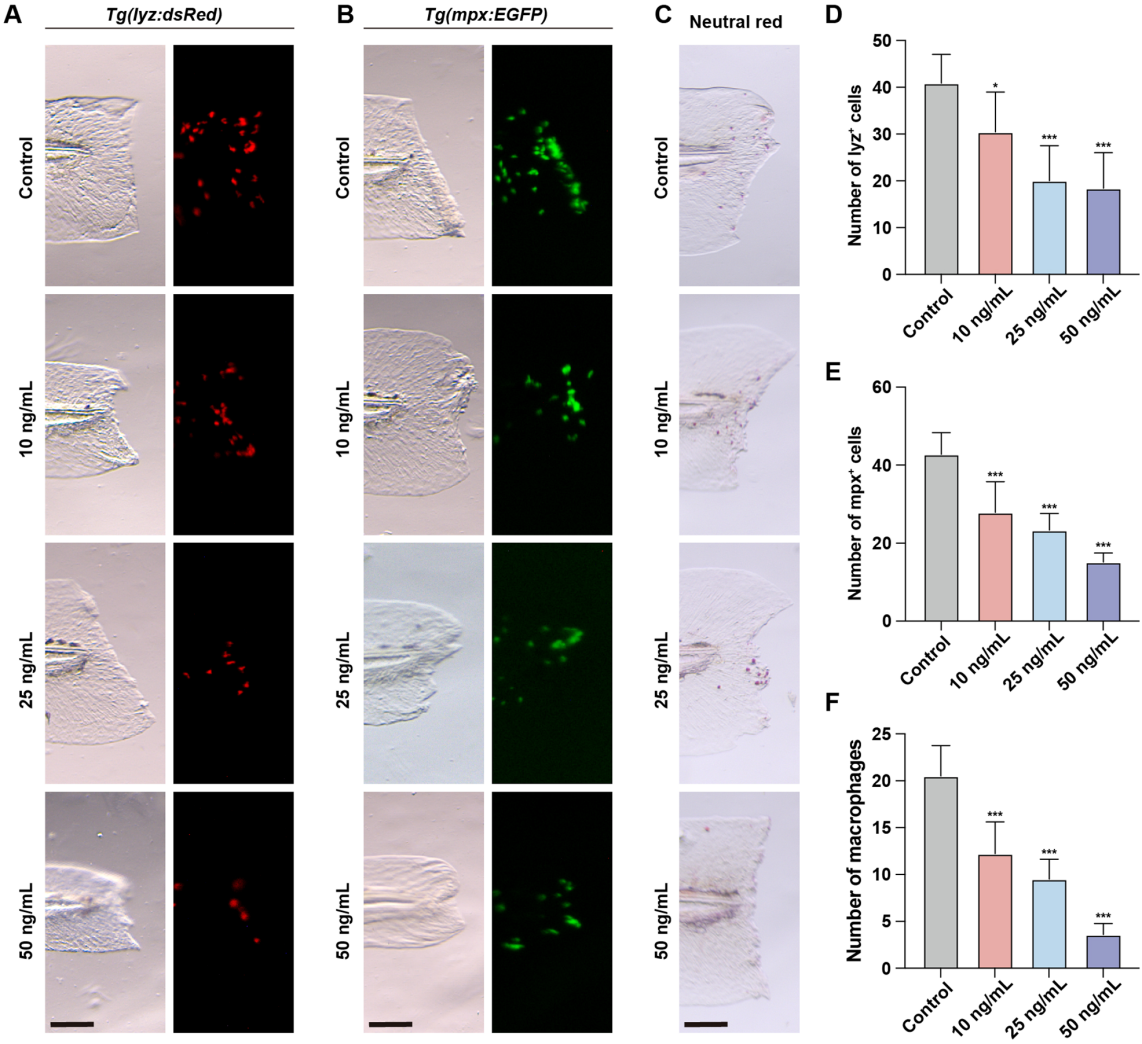
OTA impaired the immune response during zebrafish fin regeneration. **(A–C)** Representative images of control and OTA-treated *Tg(lyz:dsRed)* **(A)**, *Tg(mpx:EGFP)* **(B)** and wild-type **(C)** zebrafish larvae at 3 dpf. Embryos were imaged at 4 hours post amputation. Scale bar, 100 μm. **(D–F)** Statistical analysis of the number of neutrophils **(A, B)** and macrophages **(C)** at the wound area. One-way ANOVA-Dunnett test, *p < 0.05, ***p < 0.001. Error bar represents standard deviation.

To investigate the impact of OTA-induced immunotoxicity on immune activation following injury, we employed the zebrafish embryonic caudal fin injury model. The initial step in the immune response during fin regeneration involves the migration of neutrophils and macrophages to the injury site. These cells are responsible for clearing apoptotic or necrotic cells at the injured area and promoting inflammation. In untreated zebrafish, a rapid influx of neutrophils and macrophages to the injured area was observed after caudal fin injury (**Figures 3A–F**). However, upon OTA treatment, both neutrophil and macrophage numbers in the injured area were significantly decreased in a dose-dependent manner, indicating that OTA exposure hampers immune activation following caudal fin injury in zebrafish larvae.

### OTA induced oxidative stress in embryonic zebrafish

**Figure 4:**
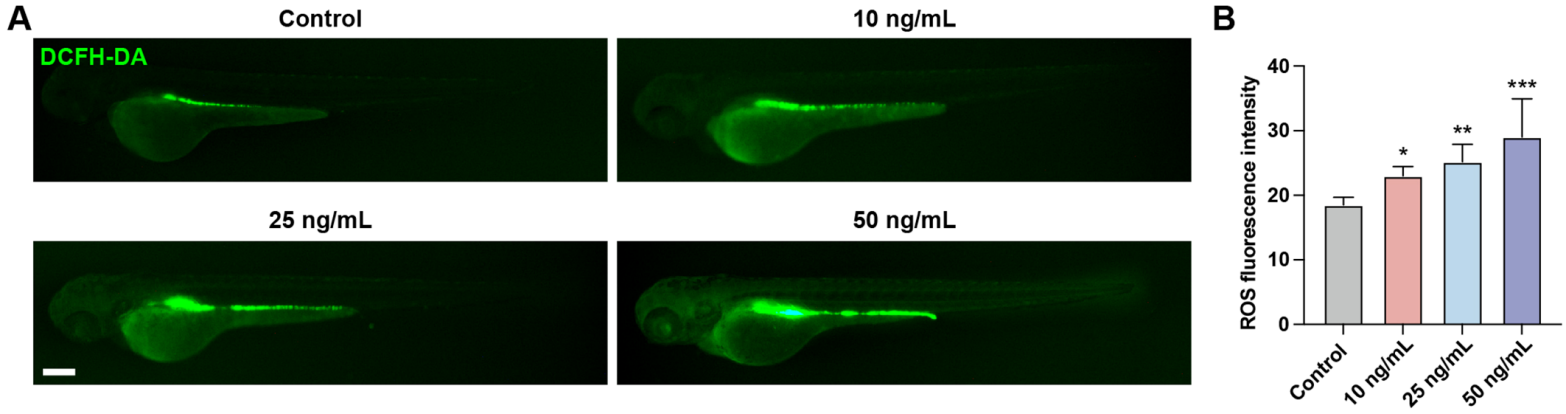
OTA induced oxidative stress in zebrafish larvae. **(A)** Representative DCFH-DA staining results of zebrafish larvae with or without OTA treatment at 3 dpf. Scale bar, 200 μm. **(B)** Statistical analysis of ROS fluorescence intensity in control and OTA-treated zebrafish larvae. One-way ANOVA-Dunnett test, *p < 0.05, **p < 0.01, ***p < 0.001. Error bar represents standard deviation.

Oxidative stress is a state of imbalance between oxidation and antioxidation in the body, resulting in a cascade of deleterious effects. Exposure to chemical toxins often leads to an elevation in reactive oxygen species (ROS) levels within the body, which induces oxidative damage to cellular biomolecules. We evaluated ROS levels in zebrafish larvae using the fluorescent probe DCFH-DA. Results showed that following OTA exposure, ROS levels were significantly increased, suggesting that OTA stimulation triggered ROS production in zebrafish larvae (**Figures 4A, B**).

### OTA induced neutrophils apoptosis in embryonic zebrafish

**Figure 5:**
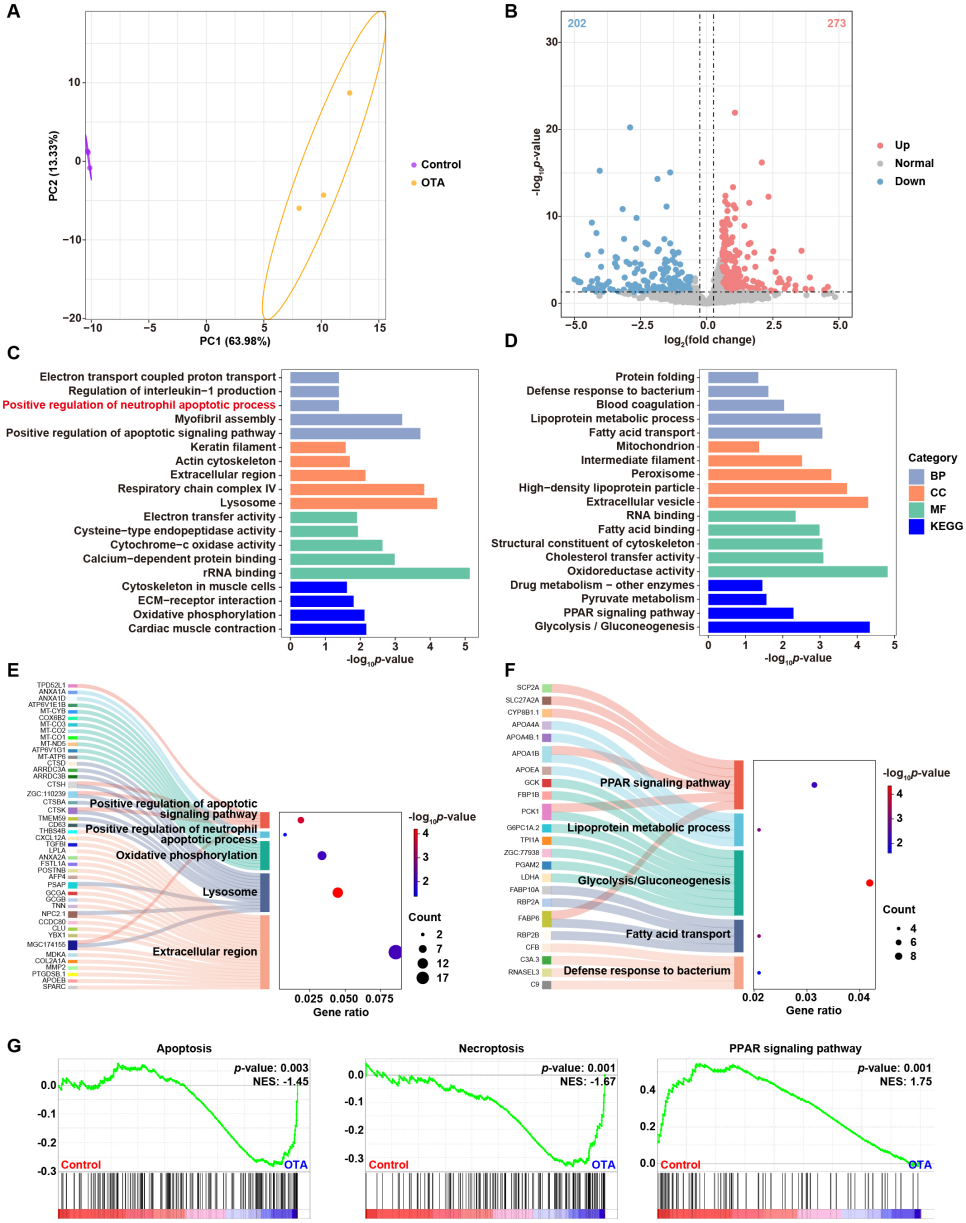
Molecular diversity of embryonic zebrafish after OTA treatment. **(A)** PCA analysis of RNA-seq results showing the heterogeneity of control and OTA-treated zebrafish embryos. **(B)** Volcano plot showing differential expressed genes between control and OTA-treated embryos. **(C, D)** Representative GO and KEGG terms of upregulated **(C)** and downregulated **(D)** genes following OTA treatment. **(E, F)** Sankey diagram of specific genes involved in the GO and KEGG terms showed in **(C, D)**. **(G)** GSEA analysis showing apoptosis, necroptosis, and PPAR signaling pathway related genes in control and OTA-treated embryos.

In order to investigate the underling mechanisms of OTA-induced immunotoxicity, we conducted RNA-seq analysis and examined the DEGs in control and OTA-treated embryos. Principal component analysis (PCA) results showed clear separation between control and OTA-treated embryos at the gene expression level (**Figure 5A**). A total of 475 DEGs were identified, including 273 up-regulated genes and 202 down-regulated genes, meeting the criteria of a fold change > 1.5 or < 0.67 and a p-value < 0.05 (**Figure 5B**). GO and KEGG analyses were performed to predict the relevant biological processes (BP), cellular components (CC), molecular functions (MF) and signaling pathways associated with these DEGs. The results revealed that up-regulated genes were mainly enriched in extracellular region, lysosome, oxidative phosphorylation, positive regulation of apoptotic signaling pathway and positive regulation of neutrophil apoptotic process (**Figure 5C**). On the other hand, down-regulated genes were mainly involved in defense response to bacterium, fatty acid transport, glycolysis/gluconeogenesis, lipoprotein metabolic process and PPAR signaling pathway (**Figure 5D**). The Sankey diagram depicted in **Figures 5E, F** illustrated the specific genes involved in the aforementioned GO and KEGG terms. Subsequently, we conducted GSEA analysis to further elucidate the biological functions associated with OTA exposure. As demonstrated in **Figure 5G**, apoptosis and necroptosis signaling pathways were significantly up-regulated following OTA treatment, while PPAR signaling pathway was down-regulated, which aligned with our GO and KEGG enrichment results. These results suggested that OTA-induced immunotoxicity primarily stems from neutrophil apoptosis induction, thereby diminishing immune response.

**Figure 6:**
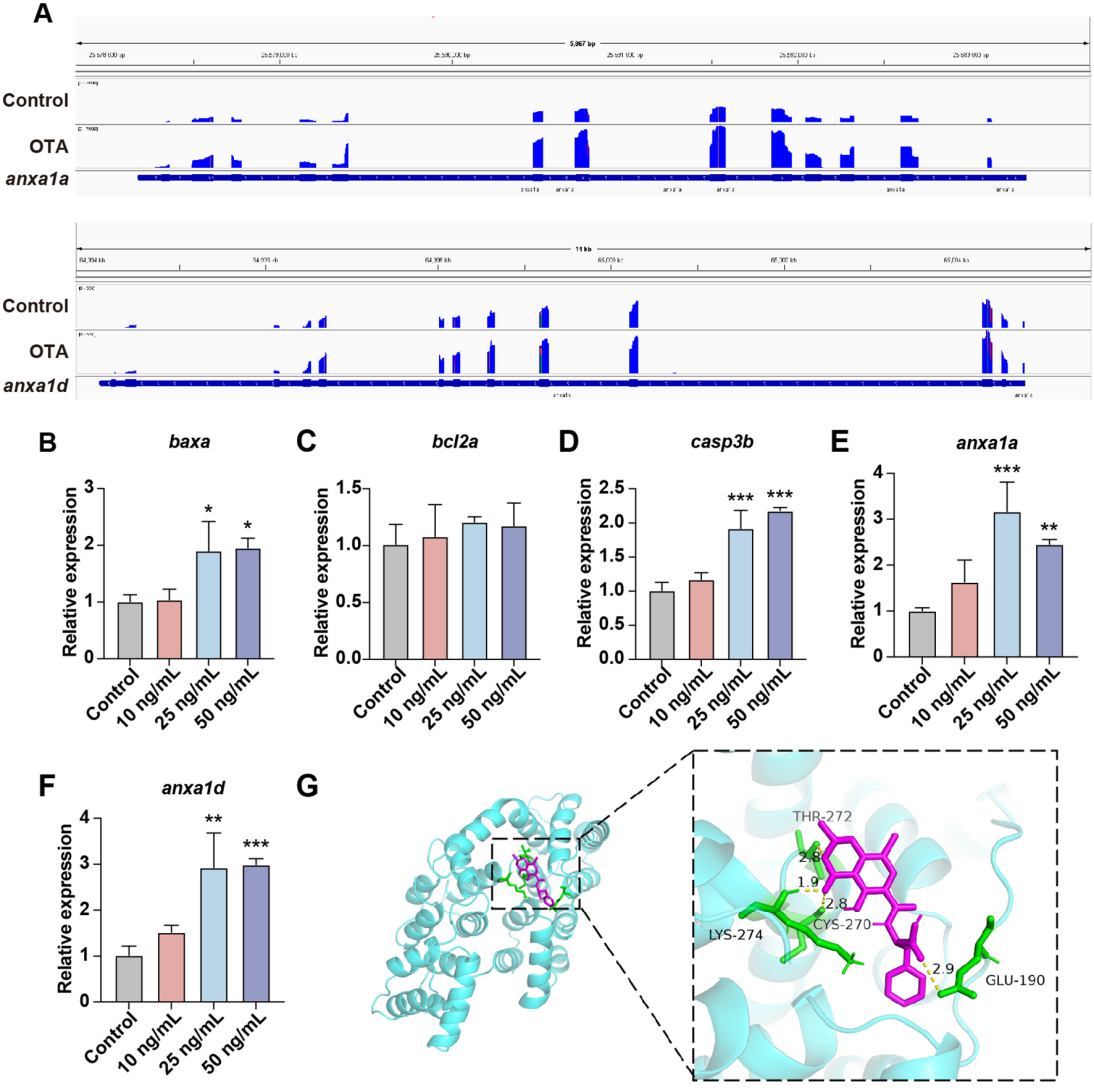
OTA induced neutrophils apoptosis in zebrafish larvae. **(A)** The expression level of *anxa1a* and *anxa1d* mRNA transcripts were shown by IGV. **(B–F)** qRT-PCR analysis showing the expression level of *baxa* **(B)**, *bcl2a* **(C)**, *casp3b* **(D)**, *anxa1a* **(E)** and *anxa1d* **(F)** in zebrafish larvae with or without OTA treatment at 3 dpf. One-way ANOVA-Dunnett test, *p < 0.05, **p < 0.01, ***p < 0.001. Error bar represents standard deviation. **(G)** Molecular interactions between OTA and ANXA1.

Subsequently, we investigated the general apoptosis events in zebrafish larvae. BAX/BCL2 and Caspase-3 play a key role in the apoptosis process. BAX predominantly induces apoptosis through the alteration of mitochondrial membrane permeability and the formation of pores, whereas BCL2 inhibits apoptosis by binding to BAX (21). Caspase-3 acts as a crucial terminal cleavage enzyme to execute the apoptotic program. Upon OTA exposure, there was a significant upregulation of *baxa* and *casp3b* expression levels (**Figures 6B–D**). Furthermore, we conducted an analysis on *anxa1a* and *anxa1d*, both of which exhibited enrichment in the aforementioned GO term associated with the positive regulation of neutrophil apoptotic process. The integrative genomic viewer (IGV) plots and qRT-PCR results revealed an upregulated expression level of *anxa1a* and *anxa1d* subsequent to OTA exposure, suggesting that OTA induces neutrophil apoptosis through the activation of *anxa1a* and *anxa1d* (**Figures 6A, E, F**).

To further substantiate whether OTA can directly bind to the ANXA1 protein, we conducted molecular docking simulations to validate the interaction between ANXA1 and OTA. The docking results showed that the binding energy of OTA and ANXA1 was -6.85 kcal/moL, showing strong binding activity (**Table 2**). OTA binds ANXA1 through the formation of hydrogen bonds at Lys274, Thr272, Cys270 and Glu190 (**Figure 6G**).This result indicated that ANXA1 was potential direct target of OTA.

**Table 2:** Molecular interactions between OTA and ANXA1.

Gene name	Protein name	Alpha Fold ID	Binding energy (kcal/moL)	H-bond number	Residues involved in H-bond formation
*ANXA1*	Annexin A1	AF-P04083-F1-v4	-6.85	4	LYS274,THR272,CYS270 and GLU190

### Aesculetin recued immunotoxicity induced by OTA exposure

**Figure 7:**
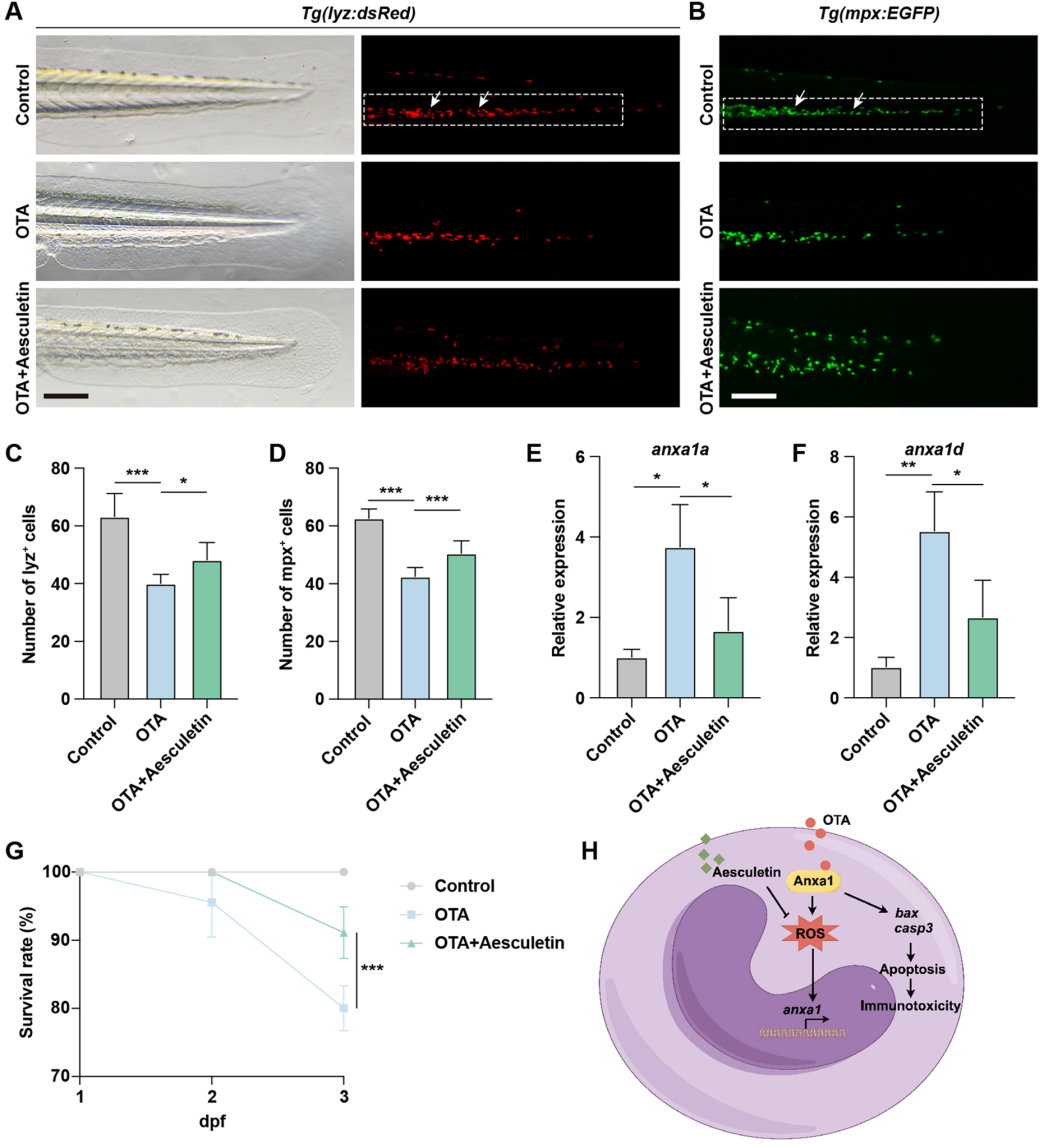
Aesculetin recued the immunotoxicity induced by OTA exposure. **(A, B)** Representative images of control, 25 ng/mL OTA and 25 ng/mL OTA + 20 μg/mL aesculetin-treated *Tg(lyz:dsRed)* **(A)** and *Tg(mpx:EGFP)* **(B)** transgenic zebrafish larvae at 3 dpf. The white dashed rectangle represents the CHT region and the white arrows represent the neutrophils. Scale bar, 100 μm. **(C, D)** Statistical analysis of the number of neutrophils in *Tg(lyz:dsRed)* **(C)** and *Tg(mpx:EGFP)* **(D)** transgenic zebrafish larvae. One-way ANOVA-Dunnett test, *p < 0.05, ***p < 0.001. Error bar represents standard deviation. **(E, F)** qRT-PCR results showing the expression level of *anxa1a* **(E)** and *anxa1d* **(F)** in control, 25 ng/mL OTA treated and 25 ng/mL OTA + 20 μg/mL aesculetin treated zebrafish larvae at 3 dpf. One-way ANOVA-Dunnett test, *p < 0.05, **p < 0.01. Error bar represents standard deviation. **(G)** Survival rate of zebrafish larvae exposed to 0, 25 ng/mL OTA and 25 ng/mL OTA + 20 μg/mL aesculetin at 2 dpf and 3 dpf. One-way ANOVA-Dunnett test, ***p < 0.001. Error bar represents standard deviation. **(H)** Schematic diagram of OTA-induced immunotoxicity in zebrafish.

The exposure to OTA resulted in elevated levels of oxidative stress in zebrafish embryos, consequently leading to neutrophil apoptosis. To investigate whether the attenuation of apoptosis levels could rescue the immunotoxicity induced by OTA, we administered aesculetin to OTA-exposed zebrafish embryos. Aesculetin has been reported for its antioxidant activity, which effectively scavenges free radicals and mitigates cell damage caused by oxidative stress, thereby protecting cells against apoptosis triggered by oxidative stress (22). Treatment with aesculetin successfully ameliorated the immunotoxicity provoked by OTA exposure (**Figures 7A–D**). At the molecular level, aesculetin treatment downregulated the elevated expression of *anxa1a* and *anxa1d* induced by OTA exposure (**Figures 7E, F**). This modulation was associated with a significant improvement in the survival rate, indicating that aesculetin may mitigate the immunotoxic effects triggered by OTA exposure (**Figure 7G**).

## Discussion

The mycotoxin OTA is a secondary metabolite produced by *Penicillium* or *Aspergillus* species, commonly found in various agricultural products. Numerous studies have reported that OTA exhibits nephrotoxic, hepatotoxic, teratogenic, neurotoxic, genotoxic, carcinogenic, and immunotoxic effects on both animals and humans, posing potential risks to public health (23). However, there is currently limited research on the impact and mechanisms of OTA on innate immunity. In this study, we demonstrated that OTA suppresses innate immunity in zebrafish by impairing embryonic development, reducing immune cell numbers, and disrupting their migration. Transcriptome analysis revealed upregulation of the *anxa1a* and *anxa1d*-mediated apoptosis pathway in neutrophils. Aesculetin effectively mitigated OTA-induced immunotoxicity, highlighting its potential as a therapeutic agent for mitigating OTA-induced immune dysfunction and offering new perspectives for the diagnosis and treatment of OTA-related toxicity (**Figure 7H**).

To investigate the developmental toxicity of OTA, we exposed zebrafish embryos to OTA at concentrations of 10 ng/mL, 25 ng/mL, and 50 ng/mL. OTA exposure resulted in a range of developmental abnormalities, including pericardial edema, shortened body length, and enlarged yolk sacs. Furthermore, zebrafish mortality and malformation rates were positively correlated with increasing OTA concentration. These findings are consistent with previous studies showing that exposure to toxic substances such as cyhalofop-butyl (24), haloxyfop-p-methyl (25) and triadimenol (26) induces similar developmental defects in zebrafish embryos as well as juveniles.

Neutrophils and macrophages are key components of the innate immune system, playing essential roles in immune defense and tissue repair. To evaluate the immunotoxic effects of OTA, we examined its impact on neutrophil and macrophage development using transgenic zebrafish lines, *Tg(lyz:dsRed)* and *Tg(mpx:EGFP)*, which specifically label neutrophils, and neutral red staining to identify macrophages. Our findings revealed a significant reduction in the number of neutrophils in the CHT following OTA treatment, while a notable decrease was observed in the number of macrophages in the dorsal head region. These results demonstrate that OTA impedes both neutrophil and macrophage development during zebrafish embryogenesis, thereby exerting immunotoxic effects on these cell types. This observation aligns with previous studies such as cyhalofop-butyl (27), haloxyfop-p-methyl (25), Niazoxanide (28), glufosinate-ammonium (29), and pyrazosulfuron-ethyl (30), all of which have been shown to inhibit neutrophil and macrophage development in zebrafish embryos. Additionally, neutrophils and macrophages play a pivotal role in the injury response, as they are among the primary cells recruited to the site of injury (31). To assess whether OTA impairs immune cell function, we used a zebrafish fin regeneration model to evaluate neutrophil and macrophage migration to injury sites. Our findings revealed a negative correlation between OTA concentration and the extent of neutrophil and macrophage migration into the tail wound area, suggesting that OTA inhibits immune responses. This finding aligns with previous studies showing that other toxicants, such as oxazolidone and nitazonide, similarly impair immune cell migration (32).

We further investigated the molecular mechanisms underlying the toxic effects of OTA on innate immunity by performing transcriptome sequencing. The results revealed an upregulation in the expression levels of *anxa1a* and *anxa1d* following OTA treatment. One advantage of using zebrafish as a model organism is that their genes and signaling pathways are highly conserved with those in humans, suggesting that these two genes may have similar functions to their human ortholog, *ANXA1*. Our data indicate that OTA directly binds to the ANXA1 protein, suggesting that OTA may function as a positive regulator of ANXA1. This interaction appears to stabilize or activate the conformation of ANXA1, thereby promoting the activation of its downstream targets BAX and CASP3, which in turn facilitates neutrophil apoptosis. Previous studies also reported that the human ANXA1 is associated with neutrophil apoptosis and its transcription leads to the synthesis of a protein with multiple inhibitory effects on inflammatory responses, promoting inflammatory cell apoptosis through transient elevation of intracellular calcium levels and activation of CASP3 (33). On the other hand, ANXA1 has been reported to enhance ROS production through the activation of the FPR1/NOX1 signaling pathway (34). Moreover, oxidative stress has been shown to further upregulate ANXA1 expression, suggesting a potential feedback mechanism (35). Therefore, our results propose that the upregulation of *anxa1a* and *anxa1d* induced by OTA may lead to neutrophil apoptosis, thereby serving as one of the mechanisms underlying its detrimental impact on innate immunity.

Aesculetin, a member of the coumarin compound family, exhibits antibacterial, anti-inflammatory, and sedative effects. Previous studies have demonstrated its antioxidant properties as well as its anti-inflammatory effects (22, 36). Additionally, it has been shown to exert hepatoprotective effects through mechanisms involving anti-apoptosis and anti-inflammation (37). Our findings revealed that aesculetin effectively counteracts OTA’s inhibitory effect on neutrophil development while restoring the up-regulated expression levels of *anxa1a* and *anxa1d* following OTA treatment. These results suggest that aesculetin protects against neutrophil apoptosis by suppressing the gene expression of *anxa1a* and *anxa1d*, thereby bolstering innate immune function and enhancing overall immunity.

In conclusion, our studies revealed the immunotoxicity of OTA and the protective effect of aesculetin using zebrafish as an animal model, thereby providing new insights into the diagnosis and treatment strategies for OTA exposure.

## Data Availability

The datasets presented in this study can be found in online repositories. The names of the repository/repositories and accession number(s) can be found below: https://www.ncbi.nlm.nih.gov/, Sequence Read Archive (SRA) database: PRJNA1167443.
